# Spectrum and Clinical Characteristics of Symptomatic and Asymptomatic Coronavirus Disease 2019 (COVID-19) With and Without Pneumonia

**DOI:** 10.3389/fmed.2021.645651

**Published:** 2021-04-01

**Authors:** Huihui Zeng, Yiming Ma, Zhiguo Zhou, Wenlong Liu, Peng Huang, Mingyan Jiang, Qimi Liu, Ping Chen, Hong Luo, Yan Chen

**Affiliations:** ^1^Department of Pulmonary and Critical Care Medicine, The Second Xiangya Hospital of Central South University, Changsha, China; ^2^Department of Respiratory Medicine, The First Hospital of Changsha, Changsha, China; ^3^Department of Respiratory Medicine, Yueyang Second People's Hospital, Yueyang, China; ^4^Department of Respiratory Medicine, Zhuzhou Central Hospital, Zhuzhou, China; ^5^Department of Respiratory and Critical Medicine, Xiangtan Central Hospital, Xiangtan, China; ^6^Department of Respiratory Medicine, The Second People's Hospital of Guilin, Guilin, China

**Keywords:** spectrum, characteristics, asymptomatic COVID-19, symptomatic, pneumonia

## Abstract

**Background:** Coronavirus disease 2019 (COVID-19), caused by severe acute respiratory syndrome coronavirus 2, has become a global pandemic. Based on symptoms, COVID-19 cases can be classified as symptomatic or asymptomatic. However, there is limited information about the differences between COVID-19 patients with and without pneumonia. Our study aimed to further discuss the spectrum and clinical characteristics of symptomatic and asymptomatic COVID-19 patients with and without pneumonia.

**Methods:** In China, all COVID-19 cases are hospitalized in designated hospitals until two continuous negative oropharyngeal swabs obtained, which allows the professional monitoring of symptoms and clinical characteristics. We stratified all COVID-19 cases in our database and evaluated clinical characteristics in different COVID-19 subgroups (symptomatic with pneumonia, symptomatic without pneumonia, asymptomatic with pneumonia, and asymptomatic without pneumonia).

**Results:** According to symptoms and laboratory and radiologic findings, COVID-19 cases were defined as symptomatic with pneumonia, symptomatic without pneumonia, asymptomatic with pneumonia, or asymptomatic without pneumonia. There were differences in the clinical characteristics and prognosis among the four groups. Both non-invasive mechanical ventilation (18, 4.2%) and invasive mechanical ventilation (11, 2.6%) were applied in only the symptomatic with pneumonia group. Likewise, extracorporeal membrane oxygenation and continuous renal replacement therapy were applied in only the symptomatic with pneumonia group. There were no differences in viral load, the durations of viral shedding, and hospitalization among the four groups.

**Conclusion:** We have defined a comprehensive spectrum of COVID-19 with and without pneumonia. The symptomatic with pneumonia group consumed more medical resources than the other groups, and extra caution and monitoring should be applied in this group. The asymptomatic COVID-19 group had a similar viral load and viral shedding duration as the symptomatic COVID-19 group.

## Introduction

Coronavirus disease 2019 (COVID-19) emerged since December 2019 in Wuhan, Hubei province, central-south China, and is an ongoing global pandemic (1). As of November 2, 2020, there were more than 45 million COVID-19 patients worldwide, and more than 1 million patients lost their lives (2). The causative pathogen has been identified as a novel enveloped RNA beta coronavirus with phylogenetic similarity to severe acute respiratory syndrome coronavirus (SARS-CoV) (3) and has been named SARS-CoV-2 by the World Health Organization. Antiviral drugs were effective for SARS-CoV-2 only *in vitro*, and a recent clinical trial of lopinavir–ritonavir, an antiviral therapy, was not associated with any benefits in COVID-19 patients (4). Because of pandemic transmission and ineffective therapeutics, early diagnosis and quarantine seem to be crucial to combat COVID-19. The spectrum of COVID-19 ranges from asymptomatic or mild, self-limiting respiratory tract illness to severe progressive pneumonia and acute respiratory distress syndrome (ARDS) (1, 5). Approximately 80% of COVID-19 patients have non-severe illness, but the asymptomatic ratio varies widely in the literatures, which could be explained by different definitions of asymptomatic COVID-19 cases and the late onset of symptoms (5–7) during the disease course. Despite inconsistencies in the asymptomatic case proportions, it is well-accepted that asymptomatic COVID-19 patients can serve as transmission sources (7–9).

SARS-CoV-2 relies on the angiotensin-converting enzyme 2 (ACE2) receptor for cellular entry, and the expression of ACE2 has been confirmed in nasal goblet cells and alveolar epithelial cells, indicating that SARS-CoV-2 can infect both the upper and lower airways (10). However, there is limited information about differences between upper airway and lower airway (pneumonia) infections in COVID-19 patients. To better understand and identify the spectrum of COVID-19, we conducted this retrospective study to discuss the clinical characteristics in different COVID-19 groups stratified by the presence or absence of symptoms and pneumonia.

## Methods

### Study Design

This study was approved and supervised by the Medical Research Ethics Committee of the Second Xiangya Hospital, Central South University. This retrospective study was performed at the Public Health Treatment Center of Changsha, People's Hospital of Junshan District, Loudi Central Hospital, People's Hospital of Lucheng District, Xiangtan Central Hospital, and People's Hospital of Yunyang District, which were designated COVID-19 hospitals in Hunan and Hubei provinces. COVID-19 patients were admitted to a designated hospital, once they were diagnosed with COVID-19. A total of 228 patients were hospitalized in Changsha, the closest neighboring capital city of Wuhan. COVID-19 was confirmed by real-time reverse transcription–polymerase chain reaction (RT-PCR) detecting SARS-CoV-2 as described previously (3). Open reading frame 1ab (ORF1ab) and nucleocapsid protein (N) were the two targeted genes simultaneously amplified and tested. Clinical samples were quantified and expressed as a cycle threshold value (Ct value), which detected ORF1ab and N as the two targeted genes. The viral load of patients' nasopharyngeal swab samples was estimated by Ct values of N gene, when RT-PCR results were considered positive at the first time. To analyze the characteristics and outcomes of symptomatic and asymptomatic COVID-19 patients with or without pneumonia, we reviewed medical records and enrolled all COVID-19 patients (*n* = 498) from the above designated hospitals who had been discharged or died before March 30.

The following medical information was obtained: demographics, symptoms during the whole course of COVID-19 (fever, cough, expectoration, dyspnea, temperature, and respiratory rate), laboratory findings (arterial blood pH, arterial blood Pao_2_, white blood cell count, neutrophils, lymphocyte count, and serum lactate dehydrogenase), chest computed tomography (CT) findings, comorbidities and concomitant diseases, duration of hospitalization, use of antivirus treatments, and concomitant treatments during admission (corticosteroids and antibiotics).

### Variables and Definitions

Temperature was examined at least three times a day, and fever was defined as an axillary temperature > 37.3°C (include 37.3°C). Chest CT was conducted every 3–5 days during hospitalization, and the classification of abnormal CT findings associated with COVID-19–related pneumonia followed those of previous research (11). Clinical improvement was defined as no fever for >3 days, the resolution of symptoms, and radiologic improvement (1). Patients were discharged after clinical improvement and the receipt of two negative continued SARS-CoV-2 RT-PCR tests with an interval of more than 24 h.

Severe COVID-19 was defined as a respiratory rate ≥30 breaths/min, blood oxygen saturation (Sao_2_) ≤93%, a Pao_2_/fraction of inspired oxygen (Fio_2_) ratio <300 mm Hg, and/or lung infiltrates in >50% of the lung field within 24–48 h (1). Patients with respiratory failure, septic shock, and/or multiple organ dysfunction/failure were defined as critical COVID-19 patients (1). All the critical patients were admitted into intensive care unit (ICU).

The duration of viral shedding for mild or moderate COVID-19 patients was defined as the time from the date of symptom onset to the date of the last negative result from two consecutive throat swab samples with an interval of more than 24 h, without positive result in subsequent test. The duration of viral shedding for asymptomatic COVID-19 patients was defined as the time from the first-time positive SARS-CoV-2 RT-PCR test to the date of the second consecutive negative RT-PCR results, with 24-h interval and without a positive subsequent test (12, 13).

According to the presence symptoms (fever, cough, expectoration, dyspnea, fatigue, muscle soreness, and headache), COVID-19 patients were divided into asymptomatic and symptomatic COVID-19 groups. Following the pneumonia guidelines from the American Thoracic Society (14), asymptomatic patients with normal chest CT findings during hospitalization were classified as the asymptomatic without pneumonia group. In contrast, asymptomatic patients with abnormal laboratory and radiologic findings during hospitalization were classified as the asymptomatic with pneumonia group. Likewise, symptomatic COVID-19 patients were classified into symptomatic with pneumonia groups and symptomatic without pneumonia groups. Symptoms, laboratory examination results, chest CT findings, and outcomes were confirmed by two independent pulmonologists.

### Statistical Analysis

Continuous variables are presented as medians [interquartile ranges (IQRs)], and categorical variables are presented as n (%). We used the Kruskal–Wallis test, Mann–Whitney *U*-test, χ^2^ test, or Fisher exact test to compare differences between groups. A two-sided α of < 0.05 was considered statistically significant. Statistical analyses were performed using SPSS software (version 23.0; SPSS Inc., Chicago, IL).

## Results

**Figure 1:**
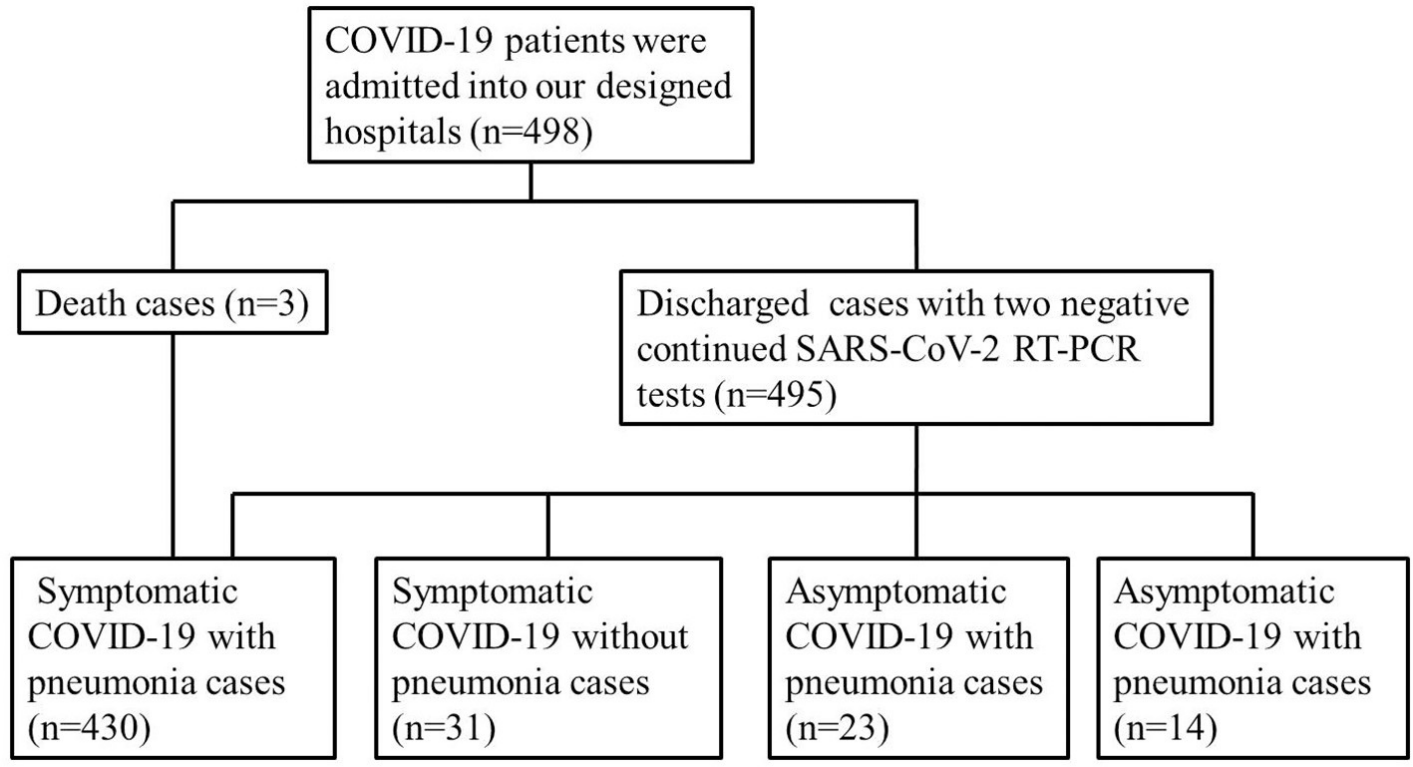
Flowchart of eligible patients.

In our study, 498 patients with confirmed COVID-19 were admitted to designated hospitals, and all of them were discharged or died before March 30. A total of 461 patients (92.6%) had symptoms and were included in the symptomatic group, whereas the other 37 patients (7.4%) were included in the asymptomatic group. Of the 461 symptomatic patients, 430 (93.3%) had abnormal laboratory and chest CT findings and were included in the symptomatic with pneumonia group, whereas the other 31 (6.2%) were included in the symptomatic without pneumonia group. Of the 37 asymptomatic COVID-19 patients, 23 (62.2%) had abnormal laboratory and chest CT findings and were classified in the asymptomatic with pneumonia group, whereas the other 14 (37.8%) were classified in the asymptomatic without pneumonia group (Figure 1). The greater frequency of pneumonia in the symptomatic group than in the asymptomatic group (*p* < 0.001 by χ^2^ test) supports the theory that symptomatic COVID-19 might be more likely to be associated with lower airway infection than with upper airway infection.

### Demographic Characteristics and Reported Symptoms

The median age of the 430 symptomatic COVID-19 with pneumonia patients was 45.0 years (IQR, 34.0–57.0 years), and 171 (39.8%) of the 430 patients were male (Table 1). The median ages of the 31 symptomatic COVID-19 without pneumonia patients, 23 asymptomatic COVID-19 with pneumonia patients, and 14 asymptomatic COVID-19 without pneumonia patients were 35.0 years (17.0–50.0 years), 48.0 years (38.0–59.0 years), and 25.0 years (11.0–51.5 years), respectively (Table 1). Statistical analysis showed that COVID-19 patients without pneumonia were younger than COVID-19 patients with pneumonia (*p* = 0.001, Table 1). Of the 23 asymptomatic pneumonia patients, only 6 (26.1%) were male, presenting a significantly lower proportion of males than females (Table 1). There were no differences in comorbidities, except for a higher frequency of hypertension (39.1%) in the asymptomatic with pneumonia group, compared with the others (Table 1).

**Table 1:** Epidemiological and clinical characteristics in symptomatic and asymptomatic COVID-19 cases.

	**Symptomatic COVID-19**		**Asymptomatic COVID-19**		
**Items**	**With pneumonia (*n* = 430)**	**Without pneumonia (*n* = 31)**	**With pneumonia (*n* = 23)**	**Without pneumonia (*n* = 14)**	***p-*value**
Age, years	45.0 (34.0–57.0)^*^	35.0 (17.0–50.0)^*^	48.0 (38.0–59.0)	25.0 (11.0–51.5)^*^	0.001
Sex, male	206 (47.9)	17 (54.8)	6 (26.1)^*^	7 (50.0)	0.175
Exposure to Wuhan	171 (39.8)	12 (38.7)	6 (26.1)	1 (7.1)^*^	0.055
Family clusters^#^	92 (47.9)^*^	13 (72.2)	8 (88.9)	5 (55.6)	0.018
Comorbidity	86 (20.0)	4 (12.9)	7 (30.4)	2 (14.3)	0.424
Influenza A or B^#^	0 (0)	0 (0)	0 (0)	0 (0)	NA
Cardiovascular disease	17 (4.0)	2 (6.5)	1 (4.3)	0 (0)	0.790
Diabetes mellitus	37 (8.6)	0 (0)	4 (17.4)	0 (0)	0.020
Hypertension	63 (14.7)	4 (12.9)	9 (39.1)^*^	1 (7.1)	0.032
COPD	12 (2.8)	1 (3.2)	0 (0)	0 (0)	0.374
Chronic liver disease	14 (3.3)	0 (0)	0 (0)	0 (0)	0.173
Chronic kidney disease	2 (0.5)	0 (0)	0 (0)	0 (0)	0.611
Malignancy	4 (0.9)	0 (0)	1 (4.3)	1 (7.1)	0.028
Cerebrovascular disease	9 (2.1)	0 (0)	0 (0)	0 (0)	0.278
Rheumatic disease	3 (0.7)	0 (0)	0 (0)	0 (0)	0.533
Signs and symptoms at admission					
Temperature at admission, °C	36.8 (36.5–37.3)^*^	36.7 (36.3–37.0)	36.6 (36.5–37.0)	36.6 (36.4–36.9)	0.021
Respiratory rate	20.0 (20.0–20.0)^*^	20.0 (20.0–20.0)	20.0 (20.0–20.0)	20.0 (18.0–20.0)	0.042
Fever	312 (72.6)	18 (58.1)	0 (0)	0 (0)	<0.001
Cough	297 (69.1)^**^	14 (45.2)	0 (0)	0 (0)	<0.001
Expectoration	178 (41.4)^**^	7 (22.6)	0 (0)	0 (0)	<0.001
Dyspnea	36 (8.4)	0 (0)	0 (0)	0 (0)	0.012
Fatigue	156 (36.3)	8 (25.8)	0 (0)	0 (0)	<0.001
Muscle soreness	56 (13.0)	5 (16.1)	0 (0)	0 (0)	0.016
Headache	34 (7.9)	1 (3.2)	0 (0)	0 (0)	0.053

*Values are presented as median (IQR) or number (percentage)*.

* *p < 0.05 compared with other groups combined*,

** *p < 0.05 compared with symptomatic COVID-19 with pneumonia cases*.

# *Data were only analyzed using cases from Changsha (n = 228); p-values were compared by Kruskal–Wallis test, χ^2^ test, Fisher exact test, or one-way analysis of variance*.

*COPD, chronic obstructive pulmonary disease; NA, not available*.

Moreover, we found that the proportion of patients reporting an exposure history to Wuhan was significantly lower in the asymptomatic without pneumonia group (7.1%) than in the other groups (Table 1), implicating that asymptomatic patients without pneumonia were more likely to be secondary cases than index cases. We have only collected family cluster history in 228 COVID-19 patients from the Public Health Treatment Center of Changsha. The results showed that symptomatic COVID-19 patients with pneumonia were less likely to be family cluster cases (47.9%, Table 1) than their counterparts.

As expected, the symptomatic with pneumonia group had a higher rate of symptoms, including a higher body temperature at admission (°C) (36.8 [IQR, 36.5–37.3]) and respiratory rate at admission (rate per min) (20 [IQR, 20–20]), cough (69.1%), and expectoration (41.4%) than the other groups (Table 1).

### Laboratory and Radiographic Findings

Routine blood tests showed a higher white blood cell count (× 10^9^ per L) (6.5 [IQR, 4.7–7.5]) and lymphocyte count (× 10^9^ per L) (2.4 [IQR, 1.7–3.0]) in the asymptomatic without pneumonia group than in the other groups (Table 2). In contrast, the symptomatic with pneumonia group had a higher frequency of lymphocytopenia (37.7%) and lower platelet count (× 10^9^ per L) (187.5 [IQR, 147.0–246.0]) than the other groups (Table 2). However, there were no differences in hemoglobin levels among groups.

**Table 2:** Laboratory findings in symptomatic and asymptomatic COVID-19 cases.

	**Symptomatic COVID-19**		**Asymptomatic COVID-19**		
**Items**	**With pneumonia (*n* = 430)**	**Without pneumonia (*n* = 31)**	**With pneumonia (*n* = 23)**	**Without pneumonia (*n* = 14)**	***p-*value**
White blood cell count, × 10^9^/L	4.6 (3.6–5.8)^*^	5.7 (4.0–6.8)	5.7 (4.8–7.8)^*^	6.5 (4.7–7.5)^*^	0.001
<4 × 10^9^/L	148 (34.4)^*^	9 (29.0)	3 (13.0)^*^	3 (21.4)	0.095
>10 × 10^9^/L	10 (2.3)	1 (3.2)	1 (4.3)	2 (14.3)	0.021
Neutrophil count, × 10^9^/L	2.9 (2.2–3.8)	3.3 (2.2–3.8)	3.6 (3.0–4.5)^*^	3.4 (2.1–4.6)	0.167
Lymphocyte count, × 10^9^/L	1.2 (0.8–1.6)^*^	1.8 (1.3–2.7)^*^	1.5 (1.3–2.0)^*^	2.4 (1.7–3.0)^*^	<0.001
Lymphocytopenia	162 (37.7)^*^	4 (12.9)^*^	3 (13.0)^*^	3 (21.4)	0.001
Hemoglobin, g/L^#^	130.0 (119.0–140.0)	129.0 (119.8–147.8)	133.0 (127.0–151.0)	125.0 (119.0–139.5)	0.431
Platelet, × 10^9^/L	187.5 (147.0–246.0)^*^	221.0 (163.5–246.0)	238.0 (217.0–305.0)^*^	232.5 (187.8–261.0)	0.002
Alanine aminotransferase, U/L	21.3 (15.0–30.4)^*^	17.7 (11.7–24.7)^*^	20.5 (16.3–23.4)	16.7 (15.1–45.1)	0.139
Aspartate aminotransferase, U/L	23.4 (18.8–31.0)	23.3 (18.7–28.0)	20.8 (17.8–24.9)^*^	24.1 (18.3–33.4)	0.249
Total bilirubin, mmol/L	11.0 (8.1–17.0)	9.6 (6.6–12.6)	10.7 (9.0–19.5)	11.2 (8.3–13.5)	0.296
Lactose dehydrogenase, U/L	177.1 (145.6–221.0)^*^	155.7 (138.5–173.5)^*^	146.2 (133.0–185.0)^*^	146.5 (128.0–198.3)	0.002
Creatinine, μmol/L	62.0 (49.0–76.0)	57.0 (38.8–75.6)	59.8 (49.9–73.0)	51.2 (29.1–66.1)	0.280
D-dimer, mg/L	0.31 (0.18–0.52)^*^	0.24 (0.12–0.37)	0.26 (0.17–0.37)	0.14 (0.09–0.24)^*^	0.009
Prothrombin time, s	12.0 (11.1–12.7)^*^	11.8 (11.1–12.8)	11.1 (10.3–12.4)^*^	11.8 (10.8–12.2)	0.066
Activated partial thromboplastin time, s	32.4 (29.2–35.6)	31.3 (30.3–33.7)	31.8 (29.6–36.8)	34.1 (32.0–36.1)	0.622
Creatine kinase, U/L	66.9 (43.0–104.9)	78.0 (58.6–94.7)	67.1 (57.0–109.0)	76.2 (50.3–104.9)	0.736
Erythrocyte sedimentation rate, mm/h^#^	44.0 (22.0–67.3)^*^	18.0 (11.0–28.5)^*^	33.0 (9.5–65.5)	19.0 (7.0–22.8)^*^	0.001
CT value of nucleic SARS-CoV-2 acid test	33.0 (27.6–35.8)	33.0 (28.8–36.3)	32.3 (29.2–36.3)	32.3 (24.6–35.9)	0.933

*Values are presented as median (IQR) or number (percentage)*.

* *p < 0.05 compared with other groups combined*.

# *Data were only analyzed using cases from Changsha (n = 228); p values were compared by Kruskal–Wallis test, χ^2^ test, Fisher exact test, or one-way analysis of variance*.

Elevated alanine aminotransferase levels (U per L) and lactose dehydrogenase (U per L) were observed in the symptomatic with pneumonia group compared with the symptomatic without pneumonia group (21.3 [IQR, 15.0–30.4] vs. 17.7 [IQR, 11.7–24.7] and 177.1 [IQR, 145.6–221.0] vs. 155.7 [IQR, 138.5–173.5], respectively) (Table 2). Compared with those in the other groups, the symptomatic with pneumonia group had a significantly higher level of d-dimer (mg per L) (0.31 [IQR, 0.18–0.52]) (Table 2). In contrast, a significantly lower level of d-dimer was observed in the asymptomatic without pneumonia group (mg per L) (0.14 [IQR, 0.09–0.24]) than in the other groups (Table 2).

We also found a higher erythrocyte sedimentation rate (mm per hour) (44.0 [IQR, 22.0–67.3]) and C-reactive protein (CRP) (U per L) (8.6 [IQR, 2.8–24.0]) in symptomatic with pneumonia group than in the other groups, whereas the asymptomatic without pneumonia group had a lower level of CRP (U per L) (1.7 [IQR, 0.3–3.0]) than the other groups (Table 2). Accordingly, among the 228 COVID-19 patients from the Public Health Treatment Center of Changsha, symptomatic patients with COVID-19 with pneumonia were more likely to have procalcitonin levels > 0.05 ng per mL (54, 28.1%) than the others (Table 2).

Antigens of influenza A and B were detected in designed hospitals, but antibodies were not detected. Interestingly, the results showed that there was no patient suffering with influenza A or B during hospitalization.

**Figure 2:**
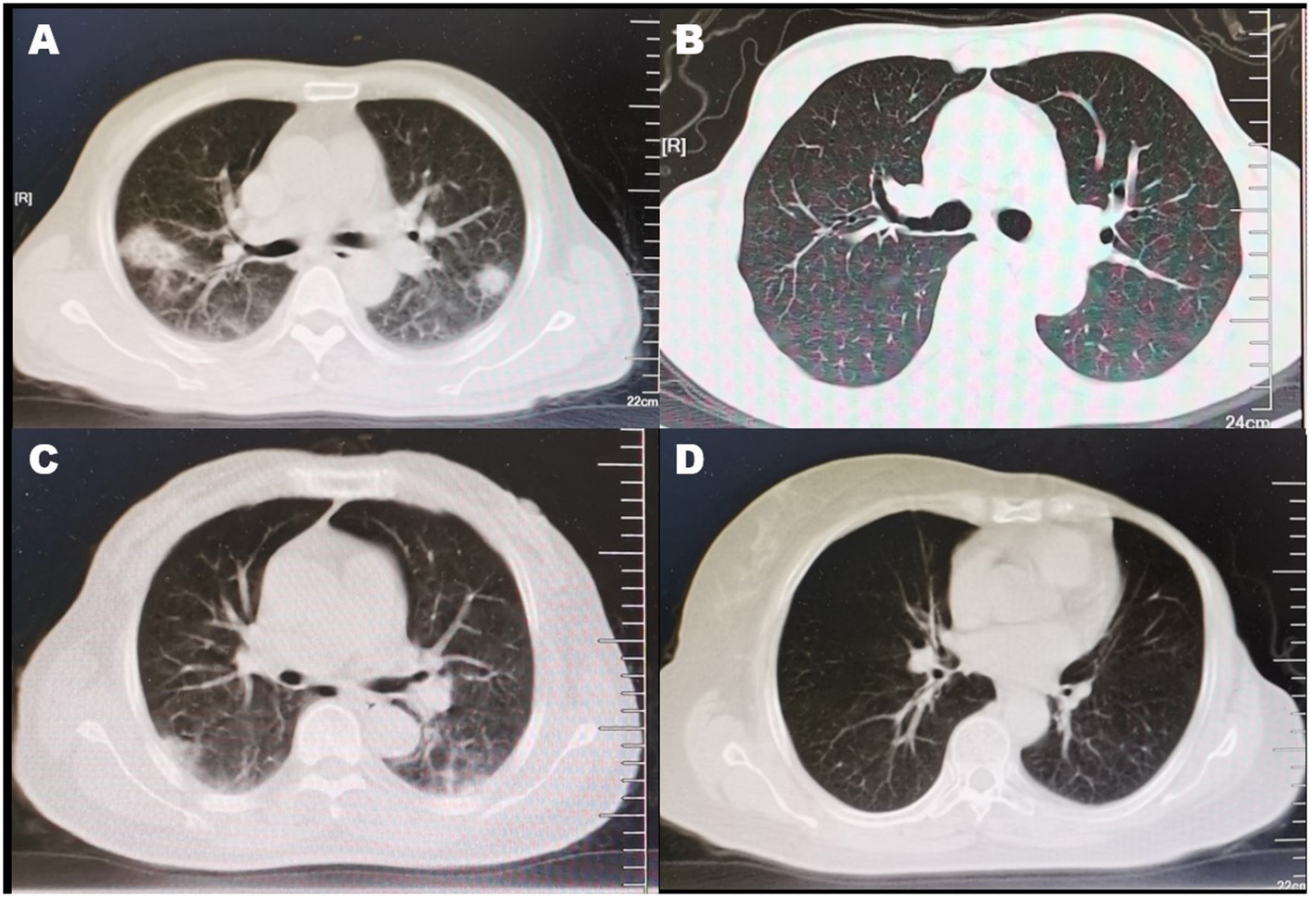
CT findings of COVID-19 patients. **(A)** Enlargement of bronchi and vascular and mixed consolidation and GGO were found in this symptomatic COVID-19 with pneumonia patient. **(B)** There was absence of abnormality in CT scan of this symptomatic COVID-19 without pneumonia patient. **(C)** Although this asymptomatic pneumonia patient had absence of symptoms, the CT scan found enlargement of bronchi and vascular and subpleural GGO and band. **(D)** This asymptomatic COVID-19 without pneumonia patient was observed with normal radiographic presentation in CT scan.

CT scan was conducted repeatedly during the hospitalization. As previously described (11), the CT findings in pneumonia patients were varied, including ground-glass opacity, consolidation, subpleural brand, and so on (Figure 2).

### Treatments and Outcomes

Excluding 10 patients who were only treated with traditional Chinese medicine in the symptomatic with pneumonia group and 1 patient who was treated with traditional Chinese medicine in the asymptomatic without pneumonia group, 487 patients received antiviral treatment, including arbidol, lopinavir/ritonavir, interferon, ribavirin, and chloroquine phosphate. In total, 254 patients (51.0%) received antibiotics, indicating the presence of secondary or concomitant bacterial infection. The symptomatic with pneumonia group had higher frequencies of antibiotic use (54.2%) and corticosteroid use (28.8) than the other groups (Table 3).

**Table 3:** Treatments and outcomes in symptomatic and asymptomatic COVID-19 cases.

	**Symptomatic COVID-19**		**Asymptomatic COVID-19**		
**Items**	**With pneumonia**	**Without pneumonia**	**With pneumonia**	**Without pneumonia**	***p-*value**
	**(*n* = 430)**	**(*n* = 31)**	**(*n* = 23)**	**(*n* = 14)**	
Treatments					
Antiviral therapy	420 (97.7)	31 (100)	23 (100)	13 (92.9)	0.865
Abidol	200 (46.5)	16 (51.6)	14 (60.9)	6 (42.9)	0.543
Lopinavir/ritonavir	310 (72.1)	24 (77.4)	10 (43.5)^*^	8 (57.1)	0.022
Interferon	259 (60.2)	24 (77.4)	14 (60.9)	8 (57.1)	0.295
Ribavirin	44 (10.2)	2 (6.5)	2 (8.7)	1 (7.1)	0.876
Chloroquine phosphate	53 (12.3)	2 (6.5)	4 (17.4)	2 (14.3)	0.635
Antibiotic therapy	233 (54.2)^*^	11 (35.5)	7 (30.4)^*^	3 (21.4)^*^	0.004
Administration of corticosteroids	124 (28.8)^*^	2 (6.5)^*^	0 (0)^*^	0 (0)^*^	<0.001
Non-invasive mechanical ventilation	18 (4.2)	0 (0)	0 (0)	0 (0)	0.121
Invasive mechanical ventilation	11 (2.6)	0 (0)	0 (0)	0 (0)	0.229
ECMO	7 (1.6)	0 (0)	0 (0)	0 (0)	0.339
CRRT	9 (2.1)	0 (0)	0 (0)	0 (0)	0.278
Clinical outcomes					
Severe cases	61 (14.2)^*^	3 (9.7)	0 (0)	0 (0)	0.011
Critical cases (admission to ICU)	46 (10.7)^*^	1 (3.2)	0 (0)	0 (0)	0.019
ARDS	18 (4.2)	0 (0)	0 (0)	0 (0)	0.121
Death	3 (0.7)	0	0	0	0.533
Duration of viral shedding, days	14.0 (9.0–21.0)	15.0 (8.8–23.8)	13.0 (7.0–19.0)	13.0 (8.3–17.8)	0.524
Duration of hospitalization, days	16.0 (11.5–24.0)	16.0 (10.0–23.0)	16.0 (11.0–22.0)	12.0 (8.8–17.0)^*^	0.234

*Values are presented as median (IQR) or number (percentage)*.

* *p < 0.05 compared with other groups combined; p-values were compared by Kruskal–Wallis test, χ^2^ test, Fisher exact test, or one-way analysis of variance*.

*ECMO, extracorporeal membrane oxygenation; CRRT, continuous renal replacement therapy; ARDS, acute respiratory distress syndrome; ICU, intensive care unit*.

Both non-invasive mechanical ventilation (18, 4.2%) and invasive mechanical ventilation (11, 2.6%) were used in only the symptomatic with pneumonia group (Table 3). Likewise, extracorporeal membrane oxygenation (ECMO) and continuous renal replacement therapy were employed in only the symptomatic with pneumonia group (Table 3). The above results indicated that patients in the symptomatic with pneumonia group suffered from more severe conditions and consumed more medical resources than those in the other groups.

All 64 severe cases occurred in symptomatic COVID-19 patients; 47 of them were critical cases and in ICU, of whom 18 developed ARDS and 3 died. Of the 430 symptomatic with pneumonia patients, 61 (14.2%) had severe COVID-19, and 46 (10.7%) were admitted to the ICU, implying that patients in the symptomatic with pneumonia group were more likely to develop severe COVID-19 and be admitted to the ICU than those in the other groups (Table 3). Interestingly, we did not find differences in the viral load, duration of viral shedding, and hospitalization among the groups (Tables 2, 3).

The follow-up of patients discharged from the Public Health Treatment Center of Changsha is ongoing. Only four of them were re-positive during 2 weeks after discharge, and all of them were from symptomatic COVID-19 with pneumonia. Moreover, continued SARS-CoV-2 RT-PCR tests showed that the contacts of the re-positive patients were not COVID-19 cases. Because the data were limited, the statistical analysis was not conducted in this study.

## Discussion

As in Middle East respiratory syndrome patients (15, 16), mounting evidence supports that presymptomatic (17, 18) or asymptomatic COVID-19 patients are infectious (6, 8, 19). In contrast with the amount of research about asymptomatic and symptomatic COVID-19 patients, there is limited research on the differences between COVID-19 patients with and without pneumonia. It is well-acknowledged that pneumonia should be confirmed by radiography findings (14). Therefore, this retrospective study describes the spectrum of COVID-19, including symptomatic COVID-19 with pneumonia, symptomatic COVID-19 without pneumonia, asymptomatic COVID-19 with pneumonia, and asymptomatic COVID-19 without pneumonia, mainly according to symptoms and CT findings. This study observed and identified characteristics and differences among the four groups, suggesting that not only symptoms, but also the presence or absence of pneumonia should be considered when evaluating the spectrum of COVID-19. Unlike some other presymptomatic or incubational cohorts (6, 17, 18), the asymptomatic COVID-19 patients in our study were discharged and monitored or evaluated according to symptoms and laboratory findings by professional healthcare workers during hospitalization, to ensure the absence of symptoms and laboratory and radiologic abnormalities during the whole disease process.

Because senior patients are vulnerable to pneumonia (14), the higher prevalence of pneumonia in the symptomatic group could be explained by the older age of the symptomatic patients. Lower levels of lymphocyte and white blood cells were observed in the symptomatic groups and were associated with better outcomes than those in the asymptomatic groups, implying that lymphocyte and white blood cells might play a protective role in COVID-19. The prevalence of hypertension was higher in the asymptomatic groups than in the symptomatic groups, suggesting that antihypertension treatment, especially ACE inhibitors might be effective for COVID-19.

A number of studies have demonstrated SARS-CoV-2 viral loads in the upper airway (19, 20) and that SARS-CoV-2 entry factors are highly expressed in upper airway cells (10). Moreover, Roman et al. (7) isolated live SARS-CoV-2 from upper airway specimens and found separate genotypes in the upper and lower airways samples. All of the above findings demonstrate that SARS-CoV-2 can infect the upper airway. Therefore, we speculate that COVID-19 without pneumonia might be only the SARS-CoV-2 upper airway infection. As expected, lower prevalence rates of severe cases and pneumonia were found in asymptomatic COVID-19 patients, and asymptomatic patients without pneumonia presented fewer abnormal laboratory findings and a lower risk of developing severe COVID-19 than symptomatic patients. The results suggested that SARS-CoV-2 infection in only the upper airway might be self-limiting, leading to a lack of symptoms and a favorable prognosis. Alba et al. (21) found targeted T-cell responses to SARS-CoV-2 in individuals not exposed to SARS-CoV-2, but possibly exposed to other coronaviruses. Therefore, we speculate that the asymptomatic COVID-19 patients without pneumonia might have been recently infected by other coronaviruses, resulting in the production of T cells targeting SARS-CoV-2, limiting the infected region and attenuating the severity of COVID-19. To save and make effective use of medicine sources, asymptomatic COVID-19 patients without pneumonia should be quarantined in primary hospitals or even Fangcang shelter hospitals (22), rather than tertiary hospitals if possible.

COVID-19 originated in Wuhan in November 2019, and exposure to Wuhan was considered important in the medical history of patients in previous studies (5, 23). The lower frequency of exposure to Wuhan in the asymptomatic without pneumonia group indicated a lower percentage of index cases in this group than in the other groups. Coronaviruses have the potential to mutate, and SARS-CoV-2 has exhibited patient-derived mutations and varying pathogenicity according to subtype (24, 25). Therefore, it is possible that SARS-CoV-2 in secondary patients has mutated, leading to decreased pathogenicity and asymptomatic COVID-19. Because of airborne transmission, family clusters play a role in SARS-CoV-2 spread (26, 27). Awareness of social distancing and personal protection caused by obvious symptoms in family members could explain the lower percentage of family clusters in the symptomatic with pneumonia group than in the other groups. On the other hand, the absence of influenza A or B in these COVID-19 patients also could be explained by the awareness of social distancing and personal protection.

Both the symptoms and laboratory findings in results support that symptomatic COVID-19 patients suffer from more severe disease than asymptomatic COVID-19 patients. A higher rate of ECMO and a higher frequency of ICU admission in the symptomatic with pneumonia group indicated not only the greater consumption of medical resources but also the need of additional monitoring in this group. In contrast, we did not find differences in viral load, viral shedding, and hospitalization durations between the symptomatic and asymptomatic groups as in previous studies (19, 28). The results suggested that asymptomatic COVID-19 might present similar pathogenicity and process of viral shedding as symptomatic COVID-19.

Asymptomatic COVID-19 with pneumonia patients also presented significant characteristics. Compared with the other patients, they are more likely to be male with hypertension and more frequently presented with lymphocytopenia and dysregulation of aminotransferase and prothrombin time. Although the asymptomatic with pneumonia subgroup showed favorable outcomes without ventilation and severe cases, statistical analysis could not find any difference between the only two pneumonia groups (symptomatic vs. asymptomatic). The limited sample size of asymptomatic with pneumonia might be a reason, and further study with larger asymptomatic with pneumonia cases is needed.

Because of a lack of significant clinical symptoms, using symptoms to screen for asymptomatic COVID-19 is difficult (17, 29, 30). In China, the Center for Disease Control and Prevention conducted SARS-CoV-2 PCR testing for all close contacts of COVID-19 patients (1). Because of the strict and comprehensive screening strategy, the prevalence of asymptomatic cases seems to be higher in our study than in other studies (17). As asymptomatic COVID-19 patients can be transmission sources (7–9), and our results showed that 7.4% of COVID-19 patients were asymptomatic, a more comprehensive screening strategy for COVID-19 is urgently needed. On the other hand, follow-up showed that there were four re-positive patients, and they were all from the symptomatic with pneumonia group. Because the data were limited, there is a need to collect more re-positive cases to discuss characteristics.

There are some limitations to this study. Because almost all the patients accepted antiviral treatment, it was possible that some presymptomatic COVID-19 patients did not develop symptomatic disease and were thus classified in the asymptomatic group. However, effective antiviral drugs targeting COVID-19 have not been identified thus far (31). Therefore, we considered the confusion of presymptomatic and asymptomatic COVID-19 patients under professional monitor during hospitalization might be rare. Because of the limited sample size and a lack of reliable evidences about Chinese traditional treatment against COVID-19, the authors did not exclude or analyze the Chinese traditional medicine–treated samples separately. Moreover, to assess the mutations and the infectivity of SARS-CoV-2, viral sequencing should be conducted in future studies.

## Conclusion

This spectrum of COVID-19 includes symptomatic COVID-19 with pneumonia, symptomatic COVID-19 without pneumonia, asymptomatic COVID-19 with pneumonia, and asymptomatic COVID-19 without pneumonia. The symptomatic with pneumonia group consumed more medical resources than the other groups, and extra caution of monitoring should be applied in this group. Asymptomatic COVID-19 case presented a similar viral load and viral shedding duration as symptomatic COVID-19 case.

## Data Availability Statement

The raw data supporting the conclusions of this article will be made available by the authors, without undue reservation.

## Ethics Statement

The studies involving human participants were reviewed and approved by the institutional ethics committee of The Second Xiangya Hospital (2020-010). Written informed consent to participate in this study was provided by the participants' legal guardian/next of kin.

## Author Contributions

Every author contributed to reviewing the paper. HZ performed, designed the work, and drafted the manuscript. YM performed the statistical analyses. ZZ, WL, PH, MJ, and QL treated and monitored COVID-19 cases. HL and PC inspired the research questions and helped to draft the manuscript. YC was the principal investigator of the study and supervised the study and preparation of the manuscript. All authors contributed to the article and approved the submitted version.

## Conflict of Interest

The authors declare that the research was conducted in the absence of any commercial or financial relationships that could be construed as a potential conflict of interest.
